# Exploring the development of academic self-concept: a qualitative study of student success among Sama Dilaut University graduates

**DOI:** 10.3389/fpsyg.2026.1853353

**Published:** 2026-07-22

**Authors:** Jantri N. Pahut, Nilo Jayoma Castulo, Hanisa U. Taupik, Rhaniza S. Ikih, Clarissa Ayangco-Derramas

**Affiliations:** 1 Department of Language Education, College of Education, Mindanao State University - Tawi-Tawi College of Technology and Oceanography, Tawi-Tawi, Philippines; 2 Department of Educational Leadership and Professional Services, College of Education, Mindanao State University - Tawi-Tawi College of Technology and Oceanography, Tawi-Tawi, Philippines; 3 College of Islamic Arts and Sciences, Mindanao State University - Tawi-Tawi College of Technology and Oceanography, Tawi-Tawi, Philippines; 4 Department of Early Childhood and Elementary Education, College of Education, Mindanao State University - Tawi-Tawi College of Technology and Oceanography, Tawi-Tawi, Philippines

**Keywords:** Academic self-concept, Bronfenbrenner's Ecological Systems Theory, cultural identity, Indigenous education, Geographically Isolated and Disadvantaged Areas (GIDA), Sama Dilaut, student success, Philippines

## Abstract

**Purpose:**

Despite the growing recognition of academic self-concept as a critical driver of student success, limited research has examined how high-achieving Indigenous students, particularly those from Geographically Isolated and Disadvantaged Areas (GIDA) communities in the Philippines, develop and sustain a positive academic self-concept in the face of systemic discrimination and socioeconomic disadvantage. Thus, this study explored the development of academic self-concept among Sama Dilaut university graduates and how they navigated the relationship between their cultural identity and their evolving identity as successful student.

**Methodology:**

A qualitative case study design was employed, involving seven Sama Dilaut university graduates selected through purposive sampling. Data were collected through in-depth semi-structured interviews conducted in English, Filipino, and Sinama. Analysis followed a four-stage process: coding, frequency counting, theming, and categorization, with the aid of ATLAS.ti version 26. Bronfenbrenner's Ecological Systems Theory served as a sensitizing framework for interpreting the findings of the study.

**Findings:**

The results revealed that academic self-concept developed through overcoming socioeconomic and cultural challenges, including discrimination, bullying, and financial constraints, while drawing on family, peer, and community support, with spiritual commitment, personal goals, and resilience serving as critical sustaining factors. Through an ecological lens, these influences operated across *microsystem* (family, peers, teachers), *mesosystem* (school-community connections), *exosystem* (scholarship policies), macrosystem [cultural values, discrimination, Indigenous Peoples' Rights Act (IPRA)], and *chronosystem* (historical marginalization). Graduates perceived their Sama Dilaut identity as an empowering force rather than a barrier, integrating cultural values, multilingual skills, and community-oriented responsibilities to achieve academic success and personal empowerment, resulting in transformative benefits, including increased confidence, leadership capabilities, social recognition, and financial stability.

**Implications:**

The findings underscore the need for enhanced community-school collaboration, expanded financial assistance programs, culturally responsive curricula aligned with the Indigenous Peoples' Rights Act (IPRA), and leadership development opportunities for Indigenous students. Policies should recognize cultural identity as an asset while addressing the systemic barriers to educational access and success.

## Introduction

Academic self-concept (ASC), which refers to how students perceive their own academic capabilities, is crucial in influencing their academic achievements and overall growth during and after their university years. A strong ASC is closely associated with better academic performance, as students who have confidence in their abilities generally achieve more. This connection is bidirectional, indicating that academic achievement also bolsters ASC over time (Guo et al., 2022; Jansen, 2017; Marsh and Scalas, 2009; Wilson et al., 2014). Students with positive ASC are often intrinsically motivated, which boosts their academic outcomes and perseverance (Jansen, 2017; Marsh and Scalas, 2009; Sunu and Baidoo-Anu, 2024; Wilson et al., 2014). ASC is linked to a propensity for lifelong learning, which is vital for success beyond the university. Those with a higher ASC exhibit increased curiosity and self-regulation, which are essential elements of lifelong learning (Deveci, 2018). A robust ASC is a predictor of higher educational aspirations and goal-setting behaviors, which are important for long-term success (Jansen, 2017; Prince and Nurius, 2014). Moreover, positive interactions with educators and peers can strengthen the ASC, thereby enhancing academic performance. For instance, teacher empathy and encouragement significantly affect students' ASC and success (Pedraja-Rejas et al., 2025).

For Indigenous students, their motivation to continue in higher education is greatly affected by academic self-efficacy (a part of self-concept) and their expectations regarding personal and academic wellbeing (Escandón-Nagel et al., 2025). Incorporating Indigenous knowledge and culturally relevant practices into educational programs has been found to boost students' academic self-concept. For instance, the inclusion of Indigenous practices in science education has led to improvements in both self-concept and academic performance, underscoring the significance of culturally responsive teaching in building confidence and engagement (Oladipupo et al., 2025). Programs that focus on cultural pride and identity, such as internships or community-based support initiatives, also enhance academic self-concept and motivation, which are crucial for retention (Craven et al., 2026; Sharrock and Lockyer, 2008). Social factors, including teacher perceptions, peer relationships, and community support, influence the academic self-concept. Positive interactions with teachers, peers, and community leaders can strengthen Indigenous students' self-concept and, consequently, their academic persistence (Prehn et al., 2021). Community-based support strategies, such as flexible course delivery and personalized interventions, have proven effective in meeting individual needs and increasing retention rates (Hearn and Funnell, 2021; Sharrock and Lockyer, 2008). Holistic approaches that cater to both academic and cultural needs are essential. For example, self-assessment tools that evaluate progress across various domains, including self-concept, provide tailored support that enhances student retention (Hearn and Funnell, 2021).

However, Indigenous students frequently encounter low self-esteem and struggle to adjust to university life, especially when transitioning from secondary to higher education (Lydster and Murray, 2019; Primus et al., 2017). They consistently face psychological challenges, such as reconciling their cultural identity with academic expectations (Payestewa-Picazo et al., 2025). Academic systems often neglect Indigenous knowledge and viewpoints, resulting in cultural isolation and systemic discrimination (da Silva, 2025; Payestewa-Picazo et al., 2025). Financial difficulties and inflexible university programs disproportionately impact Indigenous students, particularly those from regional areas or those participating in enabling programs (Nakata, 2025; Pham et al., 2025). Academic persistence is further hindered by family obligations, separation from kinship networks, and insufficient social support systems (Lydster and Murray, 2019; Pham et al., 2025). The physical and social settings of universities can adversely affect mental health and wellbeing, which, in turn, influences completion rates (Pham et al., 2025). Although self-concept is acknowledged as a factor in academic success, there is a lack of research on its specific impact on Indigenous students' outcomes in higher education (Craven et al., 2026; Marsh et al., 2023). And also, the exploration of integrating Indigenous epistemologies and traditional knowledge into academic curricula remains limited (da Silva, 2025; De Sousa and De Gois, 2017). Thus, this study investigated the development of academic self-concept among Sama Dilaut university graduates. This study seeks to answer the following questions:

- How do Sama Dilaut university graduates describe the development of their academic self-concept and its role in their academic success?
- How do Sama Dilaut graduates perceive and navigate the relationship between their cultural identity, academic success, and personal empowerment?

## Literature review

### The role of academic self-concept on academic success

Research involving high-achieving Indigenous students has shown a mutual relationship between academic self-concept and academic achievement. This connection holds true across various subjects, such as mathematics and English, at both primary and secondary education levels (Marsh et al., 2023). A strong academic self-concept enhances educational goals, motivation, and success, even in the face of systemic obstacles (Prehn et al., 2021; Prince and Nurius, 2014). Support from the community, peers, and family plays a crucial role in positively shaping Indigenous students' academic self-concept. Elements such as teacher attitudes, peer interactions, and cultural engagement enhance resilience and help counteract structural challenges (Prehn et al., 2021). Psychological attributes such as resilience, independence, and self-determination often drive Indigenous academic success. These qualities enable students to overcome difficulties and continue their higher education (Fernández et al., 2026; Nakata, 2025). Resistance to western dominance frequently serves as a psychological motivator for Indigenous students, reinforcing their academic self-concept and aiding their success (Pechenkina, 2017). A positive academic self-concept is a strong predictor of deep and self-regulated learning. Indigenous students who aim for mastery and social goals generally achieve superior educational results (McInerney and King, 2013). However, challenges such as low self-esteem and adaptation difficulties can adversely affect their academic self-concept. Implementing targeted interventions to address these issues is essential for enhancing outcomes (Primus et al., 2017). Within educational settings, Indigenous students often experience racism and discrimination, which can obstruct their academic advancement and overall health (Coombs et al., 2025; do Nascimento Moura and de Matos, 2022). Financial challenges pose a significant obstacle, as many Indigenous students come from economically disadvantaged backgrounds (Cooper et al., 2018; Cubillas, 2024). Language barriers can hinder Indigenous students' comprehension of lectures and course content, affecting their academic results (Heong et al., 2024; Marma and Yesmin, 2024). Implementing support services tailored to Indigenous students, along with mentorship programs and financial assistance, can alleviate some of these difficulties (Lydster and Murray, 2019; Nielsen et al., 2022; Payestewa-Picazo et al., 2025). Although academic self-concept is vital for success in education, research on the psychological factors contributing to the success of high-achieving Indigenous students is limited. Further studies are necessary to explore these factors and compare them with those of non-Indigenous students (Marsh et al., 2023). It is important to empirically determine the elements that reduce Indigenous students' risk of academic disengagement. While academic self-concept is considered a crucial factor, additional research is required to deepen this understanding (Bodkin-Andrews et al., 2010).

### Indigenous students' academic success and the role of cultural identity

A robust cultural identity is frequently associated with favorable academic results, such as enhanced self-esteem, motivation, and academic success. For instance, Indigenous students who possess a strong cultural identity exhibit greater intrinsic and extrinsic motivation, even if their academic identity is not as strong (Dixon and Wallace, 2024). Similarly, affirming cultural identity within classrooms encourages aspirations to pursue higher education (Dinku and Howard-Wagner, 2026). Whether identifying with Indigenous or non-Indigenous cultures, a strong cultural connection correlates with improved academic performance. Students who lack cultural identification and assertiveness are more susceptible to underachievement because of a disconnect with mainstream educational standards (Fryberg et al., 2013). Schools that offer Indigenous students opportunities to engage with their culture boost their sense of belonging and academic involvement. Key practices include Indigenous representation, culturally tailored activities, and inclusive policies (Cain and Sukhawathanakul, 2026; Prehn et al., 2025). Teachers who cultivate culturally responsive and respectful relationships with Indigenous students have a positive impact on their academic results, retention rates, and future aspirations. It is crucial for teachers to undergo professional development to enhance their cultural responsiveness (Dinku and Howard-Wagner, 2026; Kruger and Vass, 2024; Lunda et al., 2024). Integrating Indigenous knowledge and language into the curriculum increases engagement, cultural pride, and academic success. For example, applying Indigenous teaching methods in subjects such as mathematics and science has been shown to enhance conceptual understanding and cultural preservation (Klos, 2006; Mulenga et al., 2025; Zulu and Gumede, 2025). Systemic obstacles, such as Eurocentric curricula, resource shortages, and inadequate teacher training, impede the effective incorporation of Indigenous knowledge (Bogale and Alhur, 2026; Mulenga et al., 2025). The perception of institutional support for cultural development indirectly influences academic achievement by nurturing a sense of belonging and wellbeing among students. Tribal colleges, which emphasize Indigenous culture, serve as valuable examples of support for Indigenous students (Fong et al., 2021). Indigenous students frequently encounter cultural isolation, systemic discrimination, and a lack of representation in mainstream educational settings, adversely affecting their academic success and mental health (Cain and Sukhawathanakul, 2026; Payestewa-Picazo et al., 2025).

### Theoretical underpinning

Bronfenbrenner's Ecological Systems Theory (EST) provides a framework for understanding human development through the lens of interconnected environmental systems. The theory highlights the dynamic interplay between individuals and their surroundings, structured into five nested systems: *Microsystem:* This immediate setting where direct interactions take place, including family, peers, and school (Espelage, 2014; Neal and Neal, 2013). *Mesosystem:* This involves the connections between different microsystems, such as the link between family and school (Espelage, 2014). *Exosystem:* These are external environments that indirectly affect the individual, such as a parent's workplace or community resources (Chalfen, 2020). *Macrosystem:* This encompasses the wider cultural, societal, and policy contexts that influence other systems (Bizzego and Esposito, 2023; Chalfen, 2020). *Chronosystem:* This refers to the time dimension, covering life transitions and historical events that affect development (Chalfen, 2020; Neal and Neal, 2013). The theory underscores the significance of viewing development as a result of interactions across these systems, focusing on the relationship between the individual and the environment (Fulantelli et al., 2021; Tudge and Rosa, 2020). The EST stresses the importance of interactions within the microsystem (e.g., relationships with peers, expectations from teachers) and mesosystem (e.g., connections between family and school) in forming self-concept. For example, peer interactions and persistent self-concept levels have been found to affect academic performance (Collins et al., 2022; Liu and Eckert, 2014). Protective factors at various levels of the ecological system, such as family support, teacher expectations, and school climate, are vital for academic achievement. These factors correspond to the microsystem and mesosystem levels of the EST (Giovani and Petrogiannis, 2025; Murray et al., 2024). Furthermore, broader systemic influences, such as cultural and institutional policies (macrosystem), contribute to shaping educational experiences (Pinto, 2021; Renn and Smith, 2023). For Indigenous students, the cultural and systemic elements of the macrosystem, including the incorporation of Indigenous Knowledge Systems, are particularly significant. These elements can either facilitate or impede academic success, depending on their alignment with students' cultural identities and needs (Renn and Smith, 2023). Educators and policymakers can apply EST to create culturally responsive support systems that address the specific challenges Indigenous students encounter in higher education (Collins et al., 2022; Renn and Smith, 2023). By highlighting these multiple layers of influence, this theory offers a detailed perspective for investigating the different factors of academic self-concept development. The case study design focused on Sama Dilaut university graduates and examined how their social, cultural, and educational contexts influenced the development of their academic self-concept and pursuit of success amid various challenges focusing on a case study in Tawi-Tawi.

## Methodology

### Research design

This study employed a qualitative case study design to provide an in-depth exploration of the participants narratives and meanings that shape their academic journeys, to capture their voices and uncover the underlying interpretation of their educational experiences. This approach enables the exploration of multiple cases to understand the links between various factors, such as personal, social, behavioral, psychological, organizational, cultural, and environmental influences (Halkias et al., 2022). It is also valuable for providing a comprehensive, intensive description and interpretation of phenomena (Sorin-Peters, 2004). Multiple data sources and methods were used, including interviews, observations (e.g. visiting their workplace), and document review. Moreover, triangulation was employed to strengthen the research design by clarifying meanings and identifying different perceptions (de Vries, 2020; Elhakim, 2025). The case study design focused on Sama Dilaut university graduates and examined how their social, cultural, and educational contexts influenced the development of their academic self-concept and their pursuit of success despite various challenges.

### Research context

Indigenous Peoples in the Philippines have long faced discrimination and a lack of access to education, rooted in the country's neo-colonial educational system (Eduardo and Gabriel, 2021; Ocampo et al., 2021). Indigenous students often experience discrimination within educational institutions, which leads to emotional and physical harm and is a significant cause of school dropouts (Supan and Mendoza, 2023). This discrimination is linked to perceived ethnic and racial differences and is a barrier to pursuing higher education (Supan and Mendoza, 2023). Financial issues are a major barrier, as many Indigenous families cannot afford the costs associated with education (Cubillas, 2024; Eduardo and Gabriel, 2021). This economic disadvantage is compounded by the lack of basic human needs and services, such as water, food, and shelter, which affects their overall wellbeing and ability to focus on education (Yang et al., 2020). The combination of these challenges often leads to demotivation among Indigenous students, who may struggle to see the value of pursuing education when faced with so many barriers (Cubillas, 2024; Salva, 2025).

Many Indigenous communities lack the necessary educational infrastructure, including schools, trained teachers, and learning materials (Arnado and Aviles, 2023; Firdaussy et al., 2024). This is particularly challenging in remote areas, where access to resources is limited (Firdaussy et al., 2024). Many IP students are unprepared for college, with their preparedness levels varying based on their high school background and participation in academic and extracurricular activities (Vecaldo et al., 2020). Although policies such as the Indigenous Peoples' Rights Act (IPRA) of 1997 exist, their implementation is often seen as tokenistic and does not fully address the needs of Indigenous communities (Eduardo and Gabriel, 2021). Thus, there is a need for more culturally appropriate and inclusive educational policies (Ocampo et al., 2021). Providing financial support, improving infrastructure, and ensuring access to education and social services are crucial steps to improve Indigenous Peoples' educational access (Arnado and Aviles, 2023; Cubillas, 2024; Yang et al., 2020). Engaging Indigenous communities in the educational process and incorporating their voices and cultural practices can help address these challenges (Magdadaro and Sacramento, 2022).

### Research locale and participants

This research was conducted in Bongao, Tawi-Tawi, particularly across different institutional and community settings where Sama Dilaut graduates currently work. This location included local universities, government offices, and community-based organizations in which the participants were employed. Their ages ranged from early twenties to mid-forties, with five male and two female participants included in the study. All participants identified as Sama Dilaut and practiced Islam. Their educational backgrounds included degrees in education, fisheries, science-related disciplines and community development. At the time of the study, the participants occupied different professional positions, including university instructors, research assistants, laboratory personnel, community leaders, and institutional staff. Most participants came from economically disadvantaged households and experienced financial hardships throughout their educational journeys. Several participants described working while studying, relying on scholarships, or receiving support from family members and peers to continue their education despite financial difficulties. Their experiences reflect varying educational pathways, including delayed schooling, balancing employment and academic responsibilities, and navigating cultural and institutional barriers within higher education. The inclusion of participants with different professional roles in educational experiences allowed the study to capture diverse perspectives on the development of academic self-concept among Sama Dilaut graduates. These varied trajectories also provided insight into how educational success influenced participants' social mobility, leadership roles, and community engagement after graduation (Table 1).

**Table 1:** Demographic profiling.

Pseudonym	Gender	Religious affiliation	Position	Years of working experience
A	Male	Islam	Staff laboratory technician	7 years
B	Male	Islam	Faculty member	Not specified
C	Male	Islam	Sama studies staff	Not specified
D	Male	Islam	Staff V	4 months
E	Male	Islam	ROTC staff	4 years
F	Female	Islam	Community tribal leadership IP	10 years
G	Female	Islam	Science research assistant	6 years

The researchers created this table. ROTC, reserve officers' training corps; IP, Indigenous peoples.

The researchers created the interview questions and had them peer-reviewed to ensure their relevance and accuracy to the study's objectives. The validated semi-structured interview guide was evaluated by an external expert. The semi-structured interview guide asked two main questions regarding the development of academic self-concept among Sama Dilaut University graduates. The authors clarified that, in this study, 'university graduates' are defined as undergraduate students from the Sama Dilaut community who have earned their first bachelor's degree.

### Data collection and analysis

Before data collection began, ethical approval was granted by the departmental subject ethics review (approval no. 2025-18). In the interest of reflexivity, we acknowledge that the first author identifies as Sama Dilaut, the third as Sama Tandubas, and the fourth as Sama Sibutu, while the second and final authors belong to the Bisaya community and possess prior experience working with Tawi-Tawi's various Indigenous groups. Informed consent was obtained, ensuring that the participants understood the purpose of the study and their right to withdraw at any time. Data were gathered through in-depth, semi-structured interviews designed to elicit open and reflective narratives. Each interview lasted between 40 and 50 min and was conducted in a language comfortable to the participant: English, Filipino, or Sinama. The interview questions focused on key experiences, turning points, and relationships that influenced the participants' academic self-concept and cultural identity. This study adopted a systematic and reflective approach. After the interviews, the researcher transcribed the recorded conversations verbatim to capture the participants ‘statements.

The data analysis was conducted in four distinct phases. In the first phase, known as *Coding of Statements*, the interview transcripts were meticulously reviewed, and descriptive codes were assigned to key words, phrases, and sentences to encapsulate their core meanings, using both *in vivo* and researcher-generated labels. The second phase, *Frequency Counting of Codes*, involved tallying the occurrences of each code throughout the dataset to pinpoint the experiences and viewpoints most frequently shared by participants. The third phase, *Theming (Clustering the Selected Codes)*, concentrated on assembling the most frequent and conceptually pertinent codes into broader themes that illustrated recurring patterns in the participants' narratives. In the fourth phase, *Categorizing the Themes*, these themes were organized into overarching categories that mirrored the primary dimensions of the participants' lived experiences, offering a higher level of conceptual organization. Upon completing these four phases, the researcher moved on to the Interpretation of Themes, analyzing each theme in relation to the research questions, pertinent literature, and the participants' contextual realities to derive meaningful and insightful conclusions. Following manual coding, the researchers utilized ATLAS.ti version 26 to analyze the data and generate the codes and themes for comparison from the manual coding. After producing both manual and qualitative data analyses (QDA), the researchers finalized the definitive code. Through this process, the researcher converted the interview data into meaningful findings that illustrate how Sama Dilaut university graduates developed their academic self-concept. These interviews uncovered valuable insights into the challenges, successes, and transformations of Sama Dilaut learners. The researcher composed the report with clarity and precision, employing advanced grammar and language-enhancing technologies.

## Results

Table 2 summarizes the themes, codes, and frequencies that describe the key experiences and identity-related perceptions of Sama Dilaut graduates. The first category shows that their academic self-concept was shaped largely by major challenges (51 codes), including discrimination, bullying, financial limitations, transportation difficulties and cultural differences. These barriers formed the context within which students developed resilience. In response, they employed various strategies (40 codes), such as scholarships, working student arrangements, peer and family support, and personal sacrifice. Internal motivations (31 codes), including goal orientation, prioritization, and academic commitment, further strengthened students' persistence. Spiritual foundations (25 codes) and consistent family and community support (30 codes) also played essential roles in sustaining their confidence and academic direction.

**Table 2:** Summary of themes.

Category	Theme	Selected codes	Frequency	Ecological systems theory interpretation
Development of academic self-concept and its role in success	Challenges in academic journey	• Discrimination • Bullying • Financial challenges • Transportation challenges • Cultural differences	51	**Macrosystem:** societal discrimination and cultural prejudice against Indigenous groups; **Chronosystem:** historical marginalization of Sama Dilaut communities
	Strategies overcome socioeconomic challenges	• Working student • Badjao scholarship • Peer assistant • Family support • Personal sacrifice	40	**Microsystem:** family and peer support networks; **Exosystem:** scholarship policies and institutional support
	Driving force for consistent progress	• Goal oriented • Time management • Prioritization • Academic commitment • Recognition	31	**Microsystem**: personal agency, self-regulation, and goal-setting
	Spirituality driven in academic pursuit	• Faith • Moral responsibility • Belief in wisdom • Religious commitment praying	25	**Microsystem:** personal belief system and spiritual practices; **Macrosystem:** religious community values and norms
	Family and social foundational strength	• Teacher intervention • Parental guidance • Peer support • Community support • Mentorship	30	**Microsystem:** family, peer, and teacher relationships; **Mesosystem:** connections between school and community
		Sub-total	177	
**Category**	**Themes**	**Codes**	**Frequency**	
Cultural identity integration with academic success and personal empowerment	Sama Dilaut identity as an empowering force	• Identity as opportunity • Cultural strength • Cultural values • Survival strategy • Live-life lesson	27	**Macrosystem:** cultural values, community norms, and Indigenous identity as an asset
	Personal growth through educational experience	• Acculturation • Multilingual adaptability • Educational awareness • Role model • Social responsibility	29	**Microsystem:** personal development and skill acquisition; **Mesosystem:** educational experiences enabling growth
	Empowered self-concept through dual identity	• Build confidence • Sama Dilaut pride • Leadership skills • Capacity building • Knowledge	42	**Microsystem:** self-concept and personal empowerment; **Macrosystem:** cultural pride and identity affirmation
	Transformative benefits of being university	• Reciprocation • Financial stability • Job equality • Social recognition • Self-transformation	34	**Exosystem:** employment policies and economic opportunities; **Macrosystem:** social recognition and respect
	Advocacy for Social inclusion and equality	• Displacement • Livelihood struggle • Employment aspiration • Illiteracy	29	**Macrosystem:** systemic inequality and social exclusion; **Chronosystem:** historical displacement and ongoing struggles
		Sub-total	161	
		Total	338	

The researchers tabulated this table.

The second category highlights how graduates viewed the connection between their cultural identity and their academic success. Their Sama Dilaut identity served as an empowering force (27 codes) through cultural values, lived experiences, and identity-based strengths. Themes of Personal growth through educational experience (29 codes), such as multilingual adaptability and social responsibility, demonstrated how cultural and academic growth intersected. The most frequent theme, empowered self-concept through dual identity (42 codes), reflected increased confidence, leadership, and knowledge. The transformative benefits of being a university graduate (34 codes) illustrate how higher education reshapes their identities and expands their roles in the community. Advocacy for social inclusion (29 codes), including financial stability, job equality, and social recognition.

### How do Sama Dilaut university graduates describe the development of their academic self-concept and its role in their academic success?

#### Challenges in the academic journey

Many Sama Dilaut graduates report facing discrimination based on their cultural identity. This discrimination often manifests as negative stereotypes and biases from peers, which can hinder their academic progress and mental health. Bullying is another significant challenge that these students face. They often face ridicule from their classmates, which can lead to feelings of isolation and loneliness. Financial constraints are a common barrier for many Sama Dilaut students in the Philippines. They often struggle to afford basic school supplies and transportation. Moreover, access to reliable transportation is crucial for academic success in college settings. Many students face difficulties in commuting to school, which can affect their attendance and punctuality. Cultural differences can create barriers in educational settings, leading to misunderstandings and discrimination. Some graduates expressed the following views:

“I remember when I was in elementary school, they used to bully me. I still remember them always saying, ‘You're Sama, you're smelly, you don't bathe.' I felt like I didn't belong in the learning community.”

“When I was studying, sometimes when I didn't have money for transportation, I would walk from Kasulutan to our school. My shoes were already worn out, and it was extremely exhausting, I would sweat so much before even reaching school.”

“The difficulties I experienced were primarily due to a lack of support and also a lack of income.”

#### Strategies to overcome socio-economic challenges

Key strategies included working while studying, relying on family support, seeking peer assistance, applying for the Badjao scholarship, and making personal sacrifices to achieve their goals. Many Sama Dilaut graduates take jobs to support their education. This dual commitment often requires balancing work and study, which can be challenging but is necessary for success. Families play a crucial role in the academic journeys of these students. Encouragement and financial assistance from family members can significantly impact students' ability to continue their education. Support from friends and peers is also vital to the students. Graduates often rely on each other for emotional and practical support, which helps them cope with workplace challenges. The Badjao scholarship is a significant resource for many Sama Dilaut students, providing financial assistance that alleviates some of the burdens of educational expenses. Many students make personal sacrifices to achieve their educational goals. This can include postponing work opportunities or enduring hardships to remain focused on their education.

“*In terms of finances, I experienced what it was like to be a working student from an early age. I would wake up early in the morning, around 5 or 6 a.m., to sell delicacies*.”

“*This is what I did: I studied hard no matter how difficult it was. Whether it was financial hardship or challenges in my studies, I knew I always had to make sacrifices*.”

“*Fortunately, we had the ‘Badjao Grantees Scholarship.' Then my friend said, ‘I'll lend you money, or I can even pay your enrollment for you since you're also a scholar.'*”

#### Driving force for consistent progress

Many graduates express a strong focus on their goals, which serves as a motivating factor throughout their academic journey. Effective time management is crucial for balancing academic and personal commitments. Graduates often face conflicting demands from their families, communities, and academic obligations. A strong commitment to their studies was evident among graduates. Recognition from peers and educators plays a significant role in boosting confidence.

“*You cannot please everyone. If you really want to succeed in life, you have to focus on your goals. The reason I am here is because of my family and my life goals. What has helped me succeed is that I always stay focused on my goals and aspirations*.”

“*The solution is always time management. You have to manage your time… do it now, don't wait until tomorrow*.”

“*In school, if you let fear and shyness come first, you will really fall behind. You must always be diligent and hardworking*.”

#### Spiritually driven in academic pursuit

This theme encompasses religious commitment, belief in wisdom, prayer, faith, and a sense of moral responsibility to God. These spiritual elements provide a foundation for academic and personal growth. Sama Dilaut graduates express a deep commitment to their faith, which guides their actions and decisions. The participants described the importance of wisdom in academic pursuits. They believe that seeking knowledge is not just an academic endeavor but also a spiritual obligation. Prayer is a significant practice for these graduates, serving as a source of strength and guidance. Faith plays a crucial role in overcoming these challenges. Graduates feel a strong sense of moral responsibility toward their communities and cultures. They recognize that their success can inspire others and contribute positively to their heritage.

“*If you really want to make things easier, smoother, and successful, ask Allah for guidance, because nothing is impossible. As I said, nothing is impossible when you pray to Allah*.”

“*I can tell you, and I can tell others, that my experience is a testament to the power of Allah. When you remember Allah, Allah will remember you. But if you forget Allah, I am not saying that He will forget you*.”

“*When I prayed for something, He granted it to me. In my experience, this is where I truly realized that Allah, subhanahu wa ta'ala, never sleeps when you pray*.”

#### Family and social support as foundational strength

Teachers often serve as pivotal figures in their students' academic lives. They provided academic guidance and emotional support. Parents' influence is paramount in shaping graduates' educational aspirations. Friends and peers also play a crucial role in the academic success of Sama Dilaut students. They offer companionship and encouragement, which can be vital during challenging times. Peer relationships can foster a supportive environment conducive to learning. The broader community also contributes to the educational journeys of these students. Community members often encourage academic pursuits and provide the necessary resources to support them. Mentors, including teachers and community leaders, provide guidance and inspiration to students. They help students navigate their educational paths and encourage them to strive for excellence in their studies and careers.

“*I had one of the best teachers who always encouraged me. She told me, ‘Even if you are poor, don't show that you have nothing. Don't show that you are weak. You must finish your studies so you can help your family and relatives.'*”

“*Among all the support I received, the most important is from my parents. They supported me in everything, food, clothing and especially guided me in the right way*.”

“*My friend was also one of the reasons why I took up the Fisheries Program. He always encouraged me to choose Fisheries because there are many opportunities in terms of projects and research*.”

### How do Sama Dilaut graduates perceive and navigate the relationship between their cultural identity, academic success, and personal empowerment?

#### Sama Dilaut as an empowering force

Graduates view their identity as Sama Dilaut not as a limitation but as a unique opportunity to showcase their culture and heritage. This perspective allows them to leverage their cultural background as a source of strength rather than as a barrier. Sama Dilaut's cultural heritage provides a sense of pride and resilience. The values instilled in them through their culture play a significant role in their academic and professional lives. Graduates have developed survival strategies based on their cultural experiences. The experience of being Sama Dilaut imparts valuable life lessons that shape their approach to education and success. This emphasizes the importance of recognition and support from educators in fostering a sense of belonging and achievement.

“*Being Sama Dilaut is more than just a blessing. Why? Because although there are always challenges, there are also many opportunities that come into your life. Starting in college, you begin to encounter opportunities. So, being Sama Dilaut does not create problems for us*.”

“*Being Sama Dilaut was not a hindrance during my college years; it actually helped me. I remember we often had activities where we studied Badjao culture. As a Sama Dilaut student, I already had some understanding, and especially during community interviews, I could easily relate and communicate with people because we share the same background. So, it wasn't a barrier, it actually helped me more*.”

“*The opportunities that come with being Sama Dilaut are something I am grateful for. Many programs in college, like the Badjao Grant, prioritize Sama Dilaut students, which provides additional support and opportunities*.”

#### Personal growth through educational experience

The educational journey of Sama Dilaut graduates serves as a powerful vehicle for personal growth, self-realization and empowerment. Through their academic experiences, the participants discovered their inner capabilities and strengthened their self-confidence. Education also broadened their aspirations, motivating them to become professionals and to contribute positively to their communities.

“*I remember when I was presenting my paper at an international conference in Cagayan de Oro City. That's when I really felt that students are truly valued, especially when you get the chance to present at an international conference. It also made me feel more confident*.”

“*Everything in my elementary and high school years was similar, but the highlight was in college. I became active in different organizations and graduated as Cum Laude. I then realized that I have such capabilities*.”

“*As I gradually progressed to higher levels of my academic journey, I realized the value of education. I want to become a good professional in the future. That is my dream, and that is what motivates me*.”

#### Empowered self-concept through dual identity

This theme encompasses aspects such as acculturation, multilingual adaptability, educational awareness, social responsibility, and the role of being a role model for students. Participants navigate the complexities of their cultural identities while adapting to the broader societal norms. This reflects how their cultural practices and beliefs are integrated into their educational journeys, allowing them to thrive academically. The ability to communicate in multiple languages is a significant asset for Sama Dilaut graduates (Sama Badjao). Education is highly valued in the Sama Dilaut community. Graduates feel a sense of duty to uplift their communities and serve as role models for others. Many graduates aspire to become role models for the younger generation. Moreover, Sama Dilaut empowers graduates to navigate challenges, embrace opportunities, and act as catalysts for change within their communities.

“*Education is very important to me because if I say I have no allowance, I will always have none. Especially without education, you really have nothing. But if you have education, there is hope for the future. That is why, even as a Badjao, I was able to achieve being an honor student*.”

“*From my own experience, being Sama Dilaut was not a hindrance or a challenge. I know many languages, Tagalog, Sinama, Tausug, and Bisaya, so wherever I go, I am welcomed by everyone*.”

“*To support the next generation, we should encourage them to go to school and pursue education. This way, the next generation will be inspired and convinced of the importance of learning, so they will not remain ignorant*.”

#### Transformative benefits of being university graduates

Participants often experience increased recognition and respect within their communities as a result of their educational achievement. This recognition not only enhances their self-esteem but also serves as an inspiration for others in the community to pursue their passions. Attaining a university degree often leads to better job opportunities and greater financial stability for graduates. Graduates report a sense of equality in the job market, where their qualifications are recognized regardless of their cultural background. This equality fosters a sense of belonging and encourages graduates to contribute to their communities positively. The journey of education leads to profound personal growth and transformation. Graduates often feel responsible for uplifting their communities through their achievements and contributions. This commitment to community development reflects the transformative impact of education at both individual and collective levels.

“*I think for me, it's not just about change but transformation. From someone who worked as a dishwasher to someone who worked as a janitor in the classroom, from doing laundry, up until becoming a source of motivation, I have become an inspiration to other people*.”

“*Now, alhamdulillah, we have our daily needs met, and my job is permanent. Our daily needs are stable*.”

“*When you finish your education, you can become a leader regardless of your tribe because you will have the qualifications*.”

#### Advocacy for social inclusion and equality

This theme reflects the graduates' commitment to advocating for their community's rights and opportunities, emphasizing the need for social inclusion and equality in various aspects of life. Many graduates express a strong desire for equitable employment opportunities. This highlights the awareness of the systemic barriers limiting job opportunities for the Sama Dilaut community. Despite their educational achievements, illiteracy remains a significant challenge within the community, impacting both educational attainment and employment. Graduates often face livelihood struggles that hinder their ability to pursue education and gain stable employment. Displacement is another recurring issue for the Sama Dilaut, affecting their access to education and resources. Graduates are motivated to advocate for social change and inclusion. This reflects a commitment to uplifting the community and addressing barriers. These insights illustrate the challenges faced by the Sama Dilaut community and their advocacy for social inclusion and equality, emphasizing the need for systemic changes to support their educational and employment aspirations in the Philippines.

“*If you continue going to school, you will become a knowledgeable person and you will know how to handle things properly. If you stay in school, you will gain respect from others and be able to contribute to society*.”

“*My experience showed me that even in times of hardship, we were provided for. We were given support so that all of us could continue, and I realized that through education, we gain strength and guidance. Even my mother and father taught me the importance of learning because they wanted us to have the power to succeed*.”

“*It is necessary to improve employment opportunities here for our community so that Sama Dilaut students are encouraged to study. Many people finish their education but end up without jobs. This is a reality that sometimes discourages Sama Dilaut youth, which is why many of us choose to go to the sea instead*.”

## Discussions

This qualitative study examined the key experiences, relationships, and turning points that shaped the development of a positive academic self-concept among Sama Dilaut university graduates, as well as how they navigated the relationship between their cultural identity and their emerging identity as successful student.

### Microsystem influences: family, peers, teachers, and personal agency

The findings suggest that family support and peer interactions, which are considered microsystem influences, have a direct impact on academic self-concept and identity. For the participants, guidance from parents, encouragement from peers, and support from teachers were crucial for their perseverance and success. This aligns with the literature asserting that positive teacher-student relationships enhance empathy and motivation, leading to a stronger academic self-concept and improved performance (Pedraja-Rejas et al., 2025). Similarly, individuals who are academically driven and possess a high self-concept often uphold strong family values and receive emotional support (Johnson et al., 2024; Santana Vega and Feliciano García, 2011). From an EST viewpoint, microsystems also include personal traits such as goal orientation, time management abilities, and academic dedication. Students with a strong internal locus of control and learning goal orientation tend to develop a better academic self-concept, thereby enhancing their academic achievements (Albert and Dahling, 2016). Moreover, the self-regulatory manner in which participants discussed and prioritized academics reflects psychological constructs related to Indigenous perseverance, such as resilience, independence, and self-determination (Fernández et al., 2026; Nakata, 2025).

### Microsystem and mesosystem intersection: spirituality as foundational strength and school-community connections

One notable finding is how spirituality and religion affect academic self-concept. Participants began to perceive their academic pursuits as acts of devotion, considering prayer and faith in the divine as sources of guidance and strength. This operates on both the microsystem level, which involves personal belief systems, and the macrosystem level, which encompasses the values of the religious community itself. Pechenkina (2017) refers to the integration of spirituality into academic self-concept as transformational resistance, where Indigenous students use cultural and spiritual resources to reshape their identities within educational systems that have historically marginalized them. This research adds to the body of work suggesting that spiritual commitment rooted in Islamic faith can serve as both a psychological resource and a cultural guide for Sama Dilaut students facing academic challenges in the Philippines. The participants in this study effectively described the microsystem, which is the learning environment, and the mesosystem, which involves connections between multiple microsystems. These levels of analysis can be seen as links between school and community settings. Activities such as the Badjao Scholarship Program, teacher interventions, and community support exemplify these connections. These interactions primarily reinforce participants' academic self-concept by affirming cultural identities within the academic context. Community-based strategies, including informal courses and personalized student support, have proven effective in meeting the individual needs of Indigenous students (Hearn and Funnell, 2021; Sharrock and Lockyer, 2008). The findings emphasize that schools can enhance students' self-concept and perseverance by actively collaborating with Indigenous communities through scholarships, mentorship, and culturally responsive practices (Lydster and Murray, 2019; Nielsen et al., 2022).

### Macrosystem influences: cultural identity, discrimination and systemic barriers

The primary finding of this study is that graduates perceive their cultural identity as a source of strength rather than a hindrance. This operates at the macrosystem level, involving wider cultural values and societal ideologies that influence personal development. By consistently identifying as Sama Dilaut, participants were able to draw on cultural strengths, survival skills, and life lessons that supported their academic perseverance. This observation is consistent with existing research highlighting the importance of cultural identity in promoting psychological resilience and aiding college adjustment and adaptation (Yang and Gao, 2025). Similarly, first-generation Latinx college women associated family and cultural pride with academic resilience (Rocha and Coronella, 2025), while ethnic identity motivated South Asian students in Hong Kong, fostering academic success and community upliftment (Gu et al., 2019). The findings extend this literature by demonstrating that for Sama Dilaut graduates, cultural identity acts not only as a protective mechanism but also as an empowering force that reshapes their educational engagement. Cultural values, multilingual skills, and community-oriented responsibilities were crucial to the shift in academic identity (empowered self-concept through dual identity) for the participants. This is in line with Dixon and Wallace (2024) findings that cultural identity moderates the relationship between academic identity and motivation, as a stronger cultural identity offsets a lower academic identity by sustaining higher levels of academic motivation among students.

In addition, the participants identified negative influences within the macrosystem that mirrored wider societal views of Indigenous peoples, such as discrimination, bullying, and cultural bias. These influences often manifest as harmful stereotypes and discriminatory actions from peers, which can negatively impact academic success (Supan and Mendoza, 2023). In the Philippines, for instance, cultural minorities, including Indigenous Peoples, face injustices due to the persistence of neo-colonial educational practices (Eduardo and Gabriel, 2021; Ocampo et al., 2021). Despite facing discrimination, the participants demonstrated resilience by developing coping mechanisms such as pursuing education, obtaining scholarships, and receiving support from peers and family. This resilience aligns with the observations of Prehn et al. (2025), such as teacher experience, peer relationships, and community support, influencing academic self-concept. Positive engagement with teachers, peers, and community leaders can enhance Indigenous students' self-concept and academic perseverance (Prehn et al., 2021).

### Chronosystem influences: historical context and intergenerational change

The chronosystem, a crucial aspect of time, life transitions, and historical occurrences, is evident in the narratives of participants, encompassing displacement, routine adjustments to maintain their livelihoods, and intergenerational shifts. Similar to other indigenous groups, the Sama Dilaut have faced a prolonged history of marginalization, which has limited their access to education and resources (Supan and Mendoza, 2023; Yang et al., 2020). The participants' achievements signify a chronosystem transformation, marking a generational shift from marginalization to academic success. The findings also indicate that despite educational accomplishments, graduates continue to face obstacles in securing quality employment and livelihoods, while their hometowns remain largely uneducated. This highlights the need for comprehensive systemic reform and enhancement of systemic support (Magdadaro and Sacramento, 2022) to address the aspirations of Indigenous individuals pursuing higher education and employment, alongside valuing the perspectives of First Nations Peoples and recognizing cultural traditions.

### From ecological understanding to policy and practice

- *Strengthening Microsystem and Mesosystem Supports*. The findings underscore the importance of enhancing cooperation between communities and universities, which is a mesosystem intervention. For instance, universities may participate in home visits, hold dialogue sessions, and consult culturally responsive educators to create spaces that align with community needs (Magdadaro, 2022). It is essential for teachers to receive training in culturally responsive teaching methods, as teachers who establish culturally appropriate and respectful relationships with Indigenous students positively impact their academic performance, persistence, and aspirations (Dinku and Howard-Wagner, 2026; Sulanting-Sangkula et al., 2026).
- *Expanding Exosystem Resources*. Financial assistance programs aimed at Indigenous students represent a crucial growth area. The Badjao Scholarship should be expanded to offer not only monetary aid but also assistance with transportation, educational materials, and technology access. This comprehensive support reduces dropout rates and enables students to concentrate on their studies (Cubillas, 2024; Eduardo and Gabriel, 2021).
- *Addressing Macrosystem Challenges*. Policy should view cultural identity as a valuable asset rather than an obstacle while addressing the systemic barriers that lead to low educational access. To move beyond mere symbolism with Republic Act No. 8371 (1997) or the Indigenous Peoples' Rights Act of 1997, IPRA framework, and truly address students' needs, it is essential to explore how IPRA can effectively support Indigenous students through culturally responsive curricula, Indigenous representation, and the development of fully inclusive policies (Cain and Sukhawathanakul, 2026; Prehn et al., 2025). The National Commission on Indigenous Peoples (NCIP) may create collaborative platforms with educators in higher education to ensure that curricula integrate Indigenous knowledge systems (Ocampo et al., 2021).
- *Chronosystem Considerations*. Addressing the persistent challenges of community illiteracy, displacement, and daily survival requires long-term strategic approaches to ensure sustainable development. Implementing diverse initiatives aimed at fostering leadership and empowerment among Indigenous students can enhance their confidence and academic self-perception, signifying a chronosystem transformation for future generations (Lunda et al., 2024; Payestewa-Picazo et al., 2025).

## Limitations

This study had some limitations. One notable limitation is the reliance on data from a small group of Sama Dilaut university graduates, which may not fully capture the diversity of experiences among graduates from other universities or regions in the country. Consequently, future researchers should expand the sample to include graduates from multiple universities to determine whether the experiences identified in this study are consistent across different contexts. Additionally, the study focused on self-reported experiences, which may introduce subjectivity and limit the objectivity of its findings. Future research should incorporate longitudinal designs or mixed methods to examine the sustained development of the academic self-concept over time. Finally, owing to the contextual and culturally specific focus on Sama Dilaut graduates, the findings cannot be generalized to all Indigenous or marginalized student populations in the Philippines.

## Conclusion

This study investigated the development of academic self-concept among Sama Dilaut university graduates in the Philippines. Addressing the first research question, the findings reveal that academic self-concept is developed by overcoming socioeconomic and cultural challenges, including discrimination, bullying, and financial constraints, while drawing on family, peer, and community support, with spiritual commitment, personal goals, and resilience serving as critical sustaining factors across multiple ecological levels. Addressing the second research question, graduates perceived their Sama Dilaut identity as an empowering force rather than a barrier, navigating their evolving student identity by integrating cultural values, multilingual skills, and community-oriented responsibilities to achieve academic success and personal empowerment, resulting in transformative benefits, including increased confidence, leadership capabilities, social recognition, and financial stability. Through the lens of Bronfenbrenner's Ecological Systems Theory, these findings demonstrate how academic self-concept development is shaped by dynamic interactions across immediate relationships, school-community connections, institutional policies, cultural values, and historical contexts. This study contributes to the theoretical understanding of Indigenous student success by extending ecological systems theory to the Sama Dilaut context and empirically identifying factors that sustain academic persistence. Policy and practice implications include strengthening community-school collaboration, expanding financial assistance programs, implementing culturally responsive curricula aligned with the Republic Act No. 8371 or the Indigenous Peoples' Rights Act of 1997, and developing leadership opportunities for Indigenous students. Limitations include the small sample size and limited generalizability beyond the Sama Dilaut context in Tawi-Tawi. Future research should employ longitudinal designs and comparative studies across Indigenous groups in the Philippines to further understand the sustained development of academic self-concept and cultural identity integration over time, thereby informing equitable and inclusive educational policies and practices.

## Data Availability

The raw data supporting the conclusions of this article will be made available by the authors, without undue reservation.
